# Interactors and neighbors of ULK1 complex members

**DOI:** 10.1080/15548627.2024.2414386

**Published:** 2024-10-12

**Authors:** Devanarayanan Siva Sankar, Joern Dengjel

**Affiliations:** a Department of Biology, University of Fribourg, Fribourg, Switzerland; b Global Health Institute, Swiss Federal Institute of Technology Lausanne (EPFL), Lausanne, Switzerland

**Keywords:** Affinity purification, kinase, mass spectrometry, miniturbo, proteomics, proximity labeling

## Abstract

The ULK1 kinase complex plays a crucial role in autophagosome biogenesis. To identify interactors or regulators of ULK1 complex assembly influencing autophagosome biogenesis, we performed an interaction proteomics screen. Employing both affinity purification and proximity labeling of *N*- and *C*-terminal tagged fusion proteins coupled to quantitative mass spectrometry, we identified 317 high-confidence interactors or neighbors of the four ULK1 complex members, including both member-specific and common interactors. Interactions with selective macroautophagy/autophagy receptors indicate the activation of selective autophagy pathways by 90 min of nutrient starvation. Focusing on the ULK1 effector protein BAG2, a common interactor identified by both approaches, we highlight that ULK1 phosphorylates BAG2, supporting the localization of the scaffold and autophagy inducer AMBRA1 to the ER, thereby positively regulating autophagy initiation.

**Abbreviation**: AMBRA1: autophagy and beclin 1 regulator 1; ATG: autophagy related; ER: endoplasmic reticulum; HA: hemagglutinin; KD: knockdown; KO: knockout; MS: mass spectrometry; PTM: posttranslational modification; RB1CC1/FIP200: RB1 inducible coiled-coil 1; SQSTM1/p62: sequestosome 1; ULK1: unc-51 like autophagy activating kinase 1; WIPI2: WD repeat domain, phosphoinositide interacting 2.

In macroautophagy (hereafter referred to as autophagy) transient double-membraned vesicles, *i.e*. autophagosomes, are formed *de novo*. The ULK1 Ser/Thr kinase complex is critical for canonical autophagosome biogenesis and is made up of the catalytic subunit ULK1 (or its homolog ULK2), as well as of ATG13, RB1CC1/FIP200, and ATG101. Upon stress-induced autophagy, ULK1 is recruited to the ER, leading to phosphorylation of ULK1 complex members and an array of other substrates involved in autophagosome biogenesis. Several studies employed phosphoproteomic approaches to characterize ULK1 substrates relevant for autophagy regulation. Also, either affinity purification (AP) or proximity labeling (PL) coupled to mass spectrometry (MS) were successfully used to identify interactors of the ULK1 complex important for autophagy initiation.

In our recent manuscript [1], we comprehensively characterized the ULK1 complex interactome by employing both miniTurbo-based PL- and HA-based AP-MS using single expression constructs. First, CRISPR-Cas9-based knockout (KO) cell lines of genes encoding the individual members of the ULK1 complex were generated. Then we expressed the double-tag HA-miniTurbo along with the genes of interest as fusion constructs in the respective KO cells. Using quantitative interaction proteomics, we identified 317 proteins as high-confidence interactors and neighbors, of which 229 were uniquely identified by PL-MS and 89 by AP-MS (Figure 1). Of these proteins, only 16, *i.e*. 5%, are common between the two methods, indicating that both approaches are highly complementary revealing unique sets of interacting and neighboring proteins. We found that 102 proteins interact minimally with two complex members and 215 are member-specific interactors. Additionally, out of the 317 interacting proteins, only 65 proteins are known interactors, while 252 are non-characterized.

**Figure 1.**
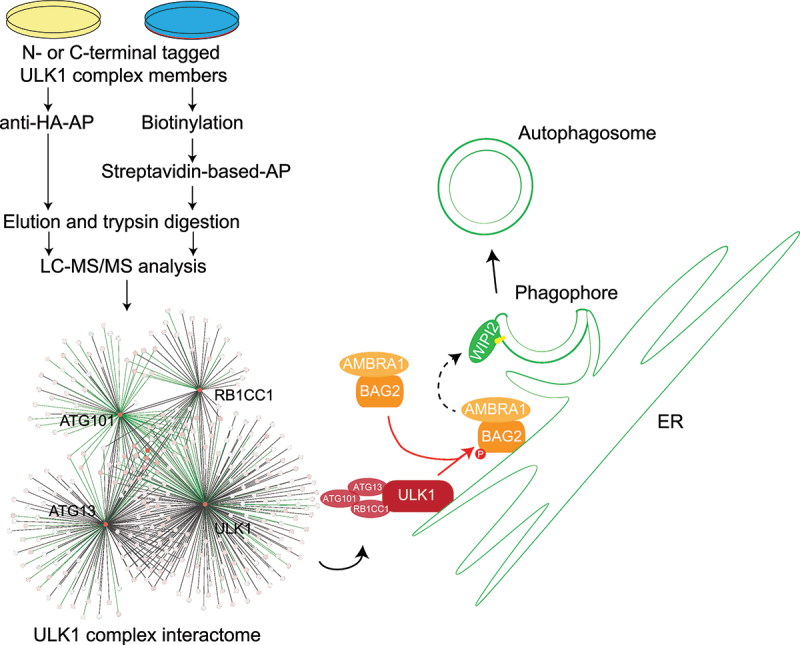
Ms-based proteomics workflow that led to the observation that ULK1 phosphorylation of its interaction partner BAG2 positively regulates autophagy initiation.

The ULK1 complex interacts with several lipid and protein kinases and phosphatases implying the formation of a macromolecular signalosome. Interestingly, selective autophagy receptors/SARs such as SQSTM1/p62, TAX1BP1, NBR1 and CCPG1 strongly interact with the ULK1 complex, showing the short-term activation of selective autophagy subpathways by 90 min of amino acid and serum starvation. This observation implies that cell stress orchestrates a combination of selective autophagy subtypes rather than inducing nonspecific bulk degradation processes. Also, the complex interaction network implies a broader function of the ULK1 complex, not only regulating autophagy induction but also its specificity and later, degradative processes.

We further studied the interactor BAG2, an HSPA/HSP70 co-chaperone which was previously shown to positively influence reticulophagy. To characterize the function of BAG2 in autophagy, we generated an interactome by AP-MS and identified increased interactions with vesicle coat proteins, ER-bound proteins and the autophagy regulator AMBRA1 in starvation conditions. Whereas both HeLa KO and shRNA-based knockdown (KD) cells lead to increased numbers of WIPI2 puncta indicating increased autophagosome biogenesis, autophagy activity is surprisingly differentially regulated: KO cells exhibit reduced autophagy flux, but transient BAG2 KD leads to increased autophagy flux. To address this discrepancy, we analyzed colocalization of SQSTM1 and LAMP2 by immunofluorescence. In KO cells, SQSTM1-LAMP2 colocalization is reduced compared to KD cells, indicating secondary, complex effects due to the chronic loss of BAG2 likely affecting vesicle trafficking as implied by its interactome.

Interactome studies also revealed a differential localization due to stress conditions. In nutrient-rich conditions, BAG2 exhibits a cytosolic/microtubular localization, whereas in starvation there is an association with endomembranes, which was demonstrated by increased binding and colocalization with the ER/Golgi-resident transmembrane protein CLCC1 (Figure 1). As BAG2 affects the localization of its client proteins, we tested its effects on AMBRA1 localization. KO cells show increased localization of AMBRA1 at the ER, independent of the stimulus, which agrees with the increased formation of WIPI2 puncta in both KO and KD cells. Using the BAG2-CLCC1 interaction as an ER localization readout, we tested for the relevance of ULK1 activity. Upon kinase inhibition or genetic ablation of ULK1, a decreased interaction and colocalization of BAG2 and CLCC1 is observed, highlighting that ULK1 controls the subcellular localization of BAG2. *In vitro* kinase assays verified that BAG2 is a direct ULK1 target and that ULK1 phosphorylates BAG2 at Ser31. Phosphomimetic and non-phosphorylatable variants of BAG2 confirmed that BAG2 and AMBRA1 localization at the ER are regulated by ULK1, its kinase activity promoting autophagosome biogenesis as indicated by increased numbers of WIPI2 puncta in cells expressing the phosphomimetic BAG2 variant. Whereas the effect of ULK1 activity and BAG2 on AMBRA1 localization is clear, open questions remain regarding the underlying molecular mechanisms. The interaction of BAG2 with CLCC1 is ULK1- and HSPA/HSP70-dependent; however, the BAG2-AMBRA1 interaction is not. Thus, additional posttranslational modifications and interactors are likely involved in the observed protein dynamics. It is also not clear if a single or several different BAG2-containing protein complexes exist at the ER membrane.

Taken together, we generated a comprehensive interactome of the ULK1 complex revealing novel interaction partners which could influence ULK1 complex assembly and autophagosome biogenesis. This dataset should serve as a resource for the autophagy research community supporting future functional studies. One new ULK1 interactor and effector protein is BAG2, which regulates the localization of AMBRA1. In nutrient-rich conditions BAG2 sequesters AMBRA1, likely on microtubules, attenuating autophagy. Upon nutrient starvation, ULK1 activation and phosphorylation of BAG2 at Ser31, BAG2 supports the localization of AMBRA1 at the ER, thereby increasing the number of autophagosome initiation sites. The functional role of the BAG2-CLCC1 interaction and a potential crosstalk with the ubiquitin-proteasome system via AMBRA1 needs to be further explored. Also, more studies are required to clarify the regulation of the PIK3C3/VPS34 complex and to investigate the functional role of BAG2 in vesicle trafficking, autophagosome maturation, and autophagosome-lysosome fusion influencing autophagosome turnover.

## References

[1] Sankar DS, Kaeser-Pebernard S, Vionnet C, et al. The ULK1 effector BAG2 regulates autophagy initiation by modulating AMBRA1 localization. Cell Rep. 2024 Aug 27;43(9):114689. doi: 10.1016/j.celrep.2024.114689 39207901

